# The role of linerixibat in treating pruritus in primary biliary cholangitis: A systematic review and meta-analysis of randomised controlled trials

**DOI:** 10.1097/MD.0000000000051203

**Published:** 2026-10-30

**Authors:** Mukesh Kumar, Vivek Kumar, Syeda Warisha, Sumet Kumar, Munazzah Khan, Mahwish Gulbahar, Savera Shabbir, Laksh Kumar, Rehma Binte Nasir, Usba Rafique, Murk Kumari Pamnani, Um E Farwa, Surhan Indra, FNU Poonam, FNU Bakhtawar, Aayush Chaulagain

**Affiliations:** a Department of Medicine, Shaheed Mohtarma Benazir Bhutto Medical College Lyari, Karachi, Pakistan; b Department of Medicine, Ziauddin Medical College, Karachi, Pakistan; c Department of Medicine, Liaquat University of Medical and Health Sciences, Jamshoro, Pakistan; d Department of Pharmacy, Shaheed Mohtarma Benazir Bhutto Medical University Institute of Pharmacy, Larkana, Pakistan; e Department of Medicine, Patan Academy of Health Sciences, Lalitpur, Nepal.

**Keywords:** IBAT inhibition, linerixibat, meta-analysis, primary biliary cholangitis, pruritus

## Abstract

**Background:**

Cholestatic pruritus affects up to 89% of patients with primary biliary cholangitis (PBC) and responds poorly to conventional treatment. Linerixibat, an oral ileal bile acid transporter inhibitor, was approved by the Food and Drug Administration in March 2026 as the first medicine indicated specifically for cholestatic-pruritus in PBC. This meta-analysis evaluated its efficacy, safety and pharmacodynamic effects.

**Methods:**

PubMed, Google Scholar, ScienceDirect, the Cochrane Library and ClinicalTrials.gov were searched from inception to April 2026 for randomized, placebo-controlled trials of linerixibat in adults with PBC and cholestatic pruritus. To avoid unit-of-analysis error, only period 1 data were used from the phase 2a crossover trial, and the shared placebo group of the multi-arm phase 2b trial was divided proportionally across active arms. Random-effects (DerSimonian–Laird) models generated mean differences (MD), standardized mean differences and risk ratios (RRs) with 95% confidence intervals. Certainty was rated using Grading of Recommendations, Assessment, Development and Evaluations (PROSPERO CRD420261381897).

**Results:**

Three trials randomizing 407 participants were included. Linerixibat reduced Worst Itch Numerical Rating Scale (WI-NRS) score versus placebo (MD −0.71; 95% confidence interval: −1.12 to −0.30; *P* = .0007; *I*^2^ = 0%), below the 2 to 3 point minimal clinically important difference. Sleep interference improved modestly (MD −0.47; *P* = .04). Responder rates were not significant at ≥2 points (RR 1.06; *P* = .49), borderline at ≥3 points (RR 1.25; *P* = .05), and nonsignificant at ≥4 points (RR 1.23; *P* = .10). Neither the PBC-40 itch domain (*P* = .14) nor 5-D Itch Scale (*P* = .28) differed significantly. Target engagement was confirmed by reduced total serum bile acids (*P* = .003) and fibroblast growth factor 19 (*P* = .0005) and increased 7α-hydroxy-4-cholesten-3-one (*P* < .00001); autotaxin was unchanged (standardized mean difference −0.20; *P* = .27). Overall adverse events were not significantly increased (RR 1.38; *P* = .06), but discontinuation (RR 2.75; *P* = .009), diarrhea (RR 3.69), abdominal pain (RR 2.80) and dyspepsia (RR 7.22) were significantly more frequent with linerixibat. Results at the licensed 40 mg twice-daily dose were concordant (WI-NRS MD −0.75; *P* = .003).

**Conclusion:**

Linerixibat produces a statistically significant but modest reduction in itch intensity below the accepted minimal clinically important difference, alongside a substantial gastrointestinal tolerability burden and roughly 3-fold increase in discontinuation. It is a mechanistically validated, licensed option for refractory cholestatic pruritus, but benefit-harm trade-offs should be discussed explicitly with patients. Grading of Recommendations, Assessment, Development and Evaluations certainty was moderate for the primary outcome.

## 1. Introduction

Primary biliary cholangitis (PBC) is a chronic autoimmune liver disease characterized by progressive cholestasis and substantial morbidity.^[1]^ Pruritus is among the most common and distressing symptoms of PBC, affecting up to 89% of patients at some point during the course of the illness.^[2,3]^ Beyond physical discomfort, cholestatic pruritus impairs daily functioning, interferes markedly with sleep and reduces health-related quality of life (HRQoL). More than half of patients with PBC and severe pruritus report significant sleep disturbance, 31% have severe depression, and their HRQoL has been described as comparable to that of people with severe Parkinson’s disease. Pruritus does not correlate with PBC disease activity, indicating that it is an independent therapeutic target and that patients with well-controlled biochemical parameters may nevertheless experience clinically substantial itch.^[4]^

Pharmacological options for cholestatic pruritus have historically been inadequate. Ursodeoxycholic acid, the standard first line therapy for PBC itself, has not been shown to relieve pruritus.^[5]^ Cholestyramine has long been recommended as first line symptomatic treatment by both American and European guidance, but it is prescribed off-label for this indication, is poorly tolerated and has limited efficacy in routine practice.^[6,7]^ Second line agents such as rifampicin, naltrexone and sertraline are likewise unlicensed for cholestatic pruritus, require monitoring for hepatotoxicity and other adverse effects, and typically produce response rates below 50%.^[6,8]^ This long-standing therapeutic gap was formally addressed only in March 2026, when the United States Food and Drug Administration approved linerixibat (Lynavoy) as the first medicine indicated specifically for the treatment of cholestatic pruritus in adults with PBC, at a recommended dose of 40 mg twice daily (BID).^[9]^ The guideline recommendations cited above predate that approval, and the evidence base underpinning it therefore merits formal quantitative synthesis.

Many patients continue to experience refractory pruritus despite stepwise treatment; invasive procedures such as nasobiliary drainage or liver transplantation have been the only route to durable relief.^[6]^ A clinical need therefore remains for targeted, well-tolerated and effective antipruritic therapy in PBC.

Linerixibat (GSK2330672) is a minimally absorbed oral ileal bile acid transporter (IBAT) inhibitor that blocks the enterohepatic circulation of bile acids in the distal ileum, promoting their fecal excretion and modifying systemic bile acid concentrations.^[10]^ Because cholestatic pruritus is thought to arise from the accumulation of bile acids and bile acid dependent pruritogens in liver, serum and skin, IBAT inhibition is a mechanistically rational strategy.^[11]^ In a phase 2a crossover study, linerixibat significantly reduced serum bile acids and itch scores relative to placebo.^[12]^ The subsequent phase 2b GLIMMER study demonstrated dose-dependent reductions in pruritus scores and identified 40 mg BID as offering the best balance of efficacy and tolerability.^[13]^ The phase 3 GLISTEN trial further characterized the therapeutic profile of linerixibat in patients with PBC and moderate-to-severe pruritus.^[4]^

Despite encouraging results from individual trials, the totality of evidence for linerixibat has not been formally synthesized. Pooling across trials is required both to obtain more precise estimates of treatment effect and to place the magnitude of that effect in the context of established thresholds for clinically meaningful change in pruritus. We therefore conducted a systematic review and meta-analysis of the efficacy and safety of linerixibat in PBC associated pruritus. The primary outcome was the mean change from baseline in itch score; secondary outcomes were pruritus responder rates, sleep interference, HRQoL itch measures, pharmacodynamic biomarkers, liver biochemistry and safety outcomes including adverse events, gastrointestinal adverse effects and treatment discontinuation.

## 2. Materials and methods

### 2.1. Protocol and reporting standards

This systematic review and meta-analysis was conducted in accordance with the Cochrane Handbook for Systematic Reviews of Interventions^[14]^ and is reported according to the Preferred Reporting Items for Systematic Reviews and Meta-Analyses (PRISMA 2020) statement.^[15]^ The protocol was prospectively registered in PROSPERO (CRD420261381897). In line with the TITAN 2025 guideline for transparent reporting of artificial intelligence in academic writing, artificial intelligence was used solely for language editing of the manuscript and was not used in the conduct of the review, study selection, data extraction or statistical analysis.^[16]^

### 2.2. Ethical approval

This study is a secondary analysis of aggregate data from previously published randomized controlled trials (RCTs) and did not involve the collection of new data from human participants or animals. Institutional review board or research ethics committee approval and informed consent were therefore not required, and the requirement was waived on the grounds that no identifiable individual patient data were accessed. Ethical approval and participant consent for each of the included trials were obtained by the original investigators and are documented in the primary publications.^[4,12,13]^

### 2.3. Literature search strategy

A comprehensive systematic literature search was conducted across PubMed, Google Scholar, ScienceDirect, the Cochrane Library (CENTRAL) and ClinicalTrials.gov from database inception through April 2026. The search strategy combined Medical Subject Headings (MeSH) terms with free text keywords. The PubMed search string used the terms “Linerixibat,” “pruritus,” “liver cirrhosis, biliary” and “primary biliary cholangitis.” Analogous search terms were adapted for the remaining databases; the full search strings are provided in Table S1, Supplemental Digital Content 1. To ensure literature saturation, the reference lists of all included trials and of relevant systematic reviews were screened manually for additional eligible studies. The search was restricted to studies published in English, with no restriction on publication date or document type.

### 2.4. Eligibility criteria and study selection

All retrieved records were screened manually and duplicates were removed. Study selection was performed independently by 2 reviewers in 2 sequential phases: screening of titles and abstracts, followed by full-text assessment of the remaining articles. Disagreements were resolved through discussion or by consultation with a third reviewer. Studies were eligible if they were RCTs, including crossover designs, that were placebo controlled and enrolled adults (≥18 years) with a confirmed diagnosis of PBC and clinically significant cholestatic pruritus, defined by a baseline Worst Itch Numerical Rating Scale (WI-NRS) or equivalent Numerical Rating Scale (NRS) score of ≥4, who received linerixibat (GSK2330672) at any dose or regimen compared with placebo. Studies were excluded if they were non-randomized or observational, or if they involved animal models, case reports, conference abstracts without full data, or narrative reviews.

### 2.5. Data extraction

For each eligible trial, 2 independent reviewers used a predesigned standardized data extraction form. Extracted data comprised study characteristics (first author, year of publication, trial phase, trial name, study design and geographic region), participant demographics (sample size, mean age, sex distribution and race or ethnicity) and disease specific baseline characteristics (duration of PBC, duration of pruritus, baseline WI-NRS or NRS itch score, pruritus severity category, concomitant ursodeoxycholic acid use and concomitant pruritus medication). Treatment details, including linerixibat dose, dosing frequency and duration of the double-blind treatment period, were also recorded.

The primary efficacy outcome was the change from baseline in WI-NRS score. Secondary outcomes were responder rates, defined as a ≥2-point, ≥3-point or ≥4-point reduction from baseline in monthly itch score; change from baseline in pruritus-related sleep interference NRS; change from baseline in the PBC-40 itch domain score; change from baseline in the 5-D Itch Scale score; and the incidence of treatment emergent and serious adverse events. Where reported, biomarker endpoints comprising total serum bile acids (TSBA), fibroblast growth factor 19 (FGF-19), serum autotaxin (ATX) activity and serum C4 (7α-hydroxy-4-cholesten-3-one) concentrations, together with alkaline phosphatase (ALP) and total bilirubin, were extracted. Discrepancies between reviewers were resolved by discussion, and primary study authors were contacted where clarification or missing data were required.

### 2.6. Handling of crossover data and shared control groups

Two features of the included trials carried a risk of unit-of-analysis error, and both were addressed explicitly before pooling. First, the phase 2a trial^[12]^ used a 2-period, 2-sequence crossover design. For all efficacy, HRQoL and pharmacodynamic biomarker outcomes we extracted and analyzed period 1 data only, that is, data collected before washout and crossover. In accordance with the Cochrane Handbook (Section 23.2.6), analyzing period 1 alone treats a crossover trial as a 2 arm parallel-group trial; this preserves randomization, removes any possibility of carryover or period effects, ensures that observations in the 2 arms are statistically independent, and permits unconfounded synthesis alongside the parallel-group trials in Review Manager. Eleven participants allocated to linerixibat and ten allocated to placebo in period 1 therefore contributed to these analyses. For safety outcomes, adverse events in the source publication are reported by treatment received across both periods rather than by period; we used these within patient totals (21 exposures per arm) because period-specific adverse event data were not recoverable from the published report. This is a residual paired data limitation that we state explicitly in the Limitations section rather than conceal.

Second, the phase 2b GLIMMER trial^[13]^ randomized participants to 5 active dose arms against a single shared placebo group. Entering the full placebo group once against each active arm would count the same control participants up to 5 times and spuriously narrow the confidence intervals (CIs). Following Cochrane Handbook guidance (Section 23.3.4) for multi-arm studies, we divided the shared placebo group approximately evenly across the active comparisons, so that each control participant contributes to the synthesis only once. The analysis-level denominators reported in the Results and displayed in each forest plot reflect this correction and are therefore smaller than the number of randomized participants.

### 2.7. Quality assessment

The methodological quality of each included RCT was appraised independently by 2 reviewers using the Cochrane Risk of Bias 2.0 (RoB 2.0; Cochrane, London, United Kingdom) tool.^[17]^ The domains assessed were the randomization process, deviations from intended interventions, missing outcome data, measurement of the outcome and selection of the reported result. Each trial received an overall judgement of low risk of bias, some concerns, or high risk of bias. Conflicting judgements were resolved by consensus or by referral to a third reviewer.

### 2.8. Certainty of evidence

The certainty of the body of evidence for each of the principal efficacy and safety outcomes was rated independently by 2 reviewers using the Grading of Recommendations, Assessment, Development and Evaluations (GRADE) approach.^[18]^ Evidence from randomized trials began at high certainty and was rated down for risk of bias, inconsistency, indirectness, imprecision and suspected publication bias. Because only 3 trials were available, publication bias could not be assessed statistically and was rated as undetected rather than absent. The complete GRADE summary-of-findings table, including the reasons for every downgrade, is presented in Table S3, Supplemental Digital Content 2.

### 2.9. Statistical analysis

Statistical analyses were performed using Review Manager (RevMan; Cochrane, London, United Kingdom) version 5.4 (The Cochrane Collaboration). For continuous outcomes, pooled estimates were expressed as mean differences (MD) with 95% CIs. Where the same construct was measured on different scales or in different units across trials, as was the case for serum ATX, the standardized mean difference (SMD) was used instead. For dichotomous outcomes, results were expressed as risk ratios (RR) with 95% CIs. A random effects model using the DerSimonian and Laird method^[19]^ was applied to all analyses. A 2-tailed *P* value of ≤.05 was considered statistically significant.

The choice of variance estimator was considered carefully given the small number of trials. The Hartung–Knapp–Sidik–Jonkman (HKSJ) adjustment is recommended where heterogeneity is present and the number of studies is small, and we evaluated it for this synthesis. We retained the standard inverse variance DerSimonian–Laird framework for 3 reasons. First, between-study heterogeneity was absent (*I*^2^ = 0%, τ^2^ = 0.00) for the primary outcome and for most secondary outcomes, including the PBC-40 itch domain, sleep interference, the 5-D Itch Scale, FGF-19, TSBA, ATX, all 3 responder thresholds, and adverse event discontinuation; under zero observed heterogeneity, the random-effects model collapses to the fixed-effect model, and HKSJ provides no variance correction. Second, HKSJ is known to behave erratically at very small study counts: with *k* = 2 the *t*-distribution has 1 degree of freedom and a critical multiplier of 12.706 against 1.96 for the standard normal distribution, producing uninformative intervals that misrepresent the precision of adequately powered trials.^[20]^ Third, the protocol prespecified analysis in RevMan 5.4, which natively implements DerSimonian–Laird estimation; applying post hoc external adjustments to selected outcomes would introduce methodological inconsistency. For the 2 outcomes with nontrivial heterogeneity and only 2 contributing studies, serum C4 and total bilirubin, neither model adjustment nor subgroup exploration is informative at *k* = 2, and we identify this explicitly as a limitation.

Statistical heterogeneity was assessed using the *I*^2^ statistic and Cochran’s *Q* test, with *I*^2^ values of <50%, 50% to 75% and >75% indicating low, moderate and high heterogeneity respectively.^[21]^ Subgroup analyses, meta-regression, and formal assessment of publication bias through funnel plots or Egger’s regression test were not performed, as the limited number of included trials renders these analyses statistically unreliable.

### 2.10. Responder and dose-specific analyses

Responder rates were defined at 3 cumulative thresholds (≥2, ≥3, and ≥4 points of reduction in WI-NRS). Because these thresholds are nested within the same patient cohort, a participant achieving a ≥4-point reduction also satisfies the ≥3-point and ≥2-point definitions. Pooling them into a single summary estimate would count individual participants up to 3 times and inflate both the apparent sample size and the precision of the estimate. Each threshold was therefore analyzed as a separate, independent meta-analysis, and no summary estimate across thresholds is reported.

The primary synthesis combined all linerixibat dose arms (20 mg once daily [QD] to 90 mg BID) against placebo in order to characterize the overall efficacy and safety of IBAT inhibition and to make use of the full dose-ranging evidence. Individual arms of the phase 2b trial enrolled only 16 to 27 participants. Because the licensed regimen is 40 mg BID, we additionally performed a prespecified dose-specific analysis restricted to arms evaluating 40 mg BID against placebo, comprising the phase 3 trial^[4]^ and the 40 mg BID arm of the phase 2b trial.^[13]^ Results of that analysis are reported alongside the primary synthesis and presented in Figures S14 to S21, Supplemental Digital Content 3.

## 3. Results

### 3.1. Study selection and characteristics

A total of 408 records were identified through the electronic databases and registers: 282 in Google Scholar, 69 in ScienceDirect, 37 in the Cochrane Library, 15 in PubMed/MEDLINE, and 5 in ClinicalTrials.gov. After removal of nine duplicates, 399 records remained for title and abstract screening, of which 383 were excluded. Sixteen records were sought for retrieval and full-text assessment. During full-text screening, 13 articles were excluded: 5 were secondary analyses of already included RCTs, 4 were systematic reviews, 2 were not original studies, and 2 did not meet the required Population, Intervention, Comparison, and Outcome criteria. Three articles satisfied all eligibility criteria and were included. The selection process is summarized in the PRISMA flow diagram (Fig. 1). Study and baseline characteristics are presented in Tables 1 and 2.

**Table 1:** Study characteristics.

Study	Trial registration	Trial name	Study design	Patient category	Total randomized	Linerixibat (n)	Placebo (n)	Dosage	Primary outcome
Hirschfield et al (2026)	NCT04950127	GLISTEN	Randomised, multicentre, double-blind, placebo-controlled, multipart, phase 3 trial	Adults (18–80 years) with proven PBC and moderate-to-severe cholestatic pruritus	238	119	119	40 mg BID	Change from baseline in monthly itch score over 24 wk
Levy et al (2023)	NCT02966834	GLIMMER	Global randomized, placebo-controlled, phase 2b dose-ranging study	Adults aged 18–80 with proven PBC and moderate-to-severe pruritus (NRS ≥ 4), including those with a score of 3 at the end of the placebo run-in	147	111 across 5 arms: 20 mg QD, n = 16 90 mg QD, n = 23 180 mg QD, n = 27 40 mg BID, n = 23 90 mg BID, n = 22	36 (single shared placebo group)	20 mg QD, 90 mg QD, 180 mg QD, 40 mg BID, 90 mg BID	Dose response of linerixibat on pruritus via mean change from baseline in monthly worst daily itch score at week 16
Hegade et al (2017)	NCT01899703	BAT117213	Phase 2a randomized, double-blind, placebo-controlled, 2-period crossover study	Adults aged 18–75 with proven or likely PBC and ongoing pruritus, maintained on stable UDCA therapy for at least 8 wk	21	11 (period 1)	10 (period 1)	45–90 mg BID	Safety and tolerability of oral GSK2330672 administered for 14 d

For Hegade et al (2017), the sample sizes shown are the period 1 denominators used in the meta-analysis of efficacy and biomarker outcomes; 22 participants were randomized in total, and 21 received treatment. For Levy et al (2023), the single placebo group of 36 participants was divided across the 5 active arms in the meta-analysis so that no control participant is counted more than once.

BID = twice daily, NRS = Numerical Rating Scale, PBC = primary biliary cholangitis, QD = once daily, UDCA = ursodeoxycholic acid.

**Table 2:** Baseline characteristics.

Variable	Hirschfield 2026 Linerixibat (n = 119)	Hirschfield 2026 Placebo (n = 119)	Levy 2023 20 mg QD (n = 16)	Levy 2023 90 mg QD (n = 23)	Levy 2023 180 mg QD (n = 27)	Levy 2023 40 mg BID (n = 23)	Levy 2023 90 mg BID (n = 22)	Levy 2023 Placebo (n = 36)	Hegade 2017 Linerixibat (n = 22)	Hegade 2017 Placebo (n = 22)
Age, yr, mean (SD)	54.7 (11.19)	57.0 (10.96)	58.5 (7.35)	52.5 (12.32)	58.9 (11.10)	55.6 (11.23)	56.2 (11.32)	54.4 (11.06)	52.9 (10.6)	52.9 (10.6)
Female, n (%)	113 (95)	113 (95)	16 (100)	21 (91)	25 (93)	22 (96)	20 (91)	34 (94)	19 (86)	19 (86)
Duration of PBC, mean (SD) or median (IQR)	55.1 (19–94)	67.8 (28–117)	9.4 (6.0)	7.2 (6.0)	10.7 (9.3)	6.7 (6.4)	7.3 (5.4)	7.5 (5.5)	5 (4.8)	5 (4.8)
Duration of pruritus, mean (SD) or median (IQR)	65.6 (31–121)	79.6 (33–125)	9.0 (6.5)	6.7 (6.0)	11.5 (10.7)	6.1 (7.2)	7.4 (5.1)	7.9 (8.6)	–	–
ALP, IU/L (Hirschfield, Hegade) or × ULN (Levy)	172.50 (106.50–327.50)	160.50 (116.50–294.50)	2.0 (2.83)	1.7 (1.12)	1.4 (1.06)	1.4 (0.72)	1.6 (1.64)	1.6 (1.24)	264 (174.1)	264 (174.1)
Total bilirubin, µmol/L (Hirschfield, Hegade) or × ULN (Levy)	9.00 (5.65–13.45)	9.50 (6.25–15.00)	0.57 (0.369)	0.62 (0.312)	0.54 (0.244)	0.47 (0.162)	0.55 (0.341)	0.60 (0.347)	12.2 (5.5)	12.2 (5.5)
Serum bile acids, µmol/L, mean (SD)	34.58 (38.57)	32.34 (44.60)	13.0 (13.8)	33.7 (55.8)	26.6 (36.5)	18.6 (21.8)	19.3 (31.0)	21.5 (31.4)	48.6 (68.7)	48.6 (68.7)
Receiving UDCA, n (%)	114 (96)	117 (98)	14 (88)	22 (96)	27 (100)	21 (91)	21 (95)	32 (89)	19 (90.4)	19 (90.4)
Pruritus severity, mild (<4), n (%)	–	–	3 (19)	5 (22)	7 (26)	7 (30)	2 (9)	11 (31)	–	–
Pruritus severity, moderate (4–<7), n (%)	51 (43)	48 (40)	12 (75)	11 (48)	13 (48)	13 (57)	13 (59)	14 (39)	–	–
Pruritus severity, severe (7–10), n (%)	68 (57)	71 (60)	1 (6)	7 (30)	7 (26)	3 (13)	7 (32)	11 (31)	–	–
Concomitant cholestyramine use, n (%)	7 (6)	9 (8)	2 (13)	4 (17)	9 (33)	9 (39)	9 (41)	9 (25)	–	–
All pruritus medications, n (%)	33 (28)	38 (32)	4 (25)	6 (26)	4 (15)	7 (30)	6 (27)	15 (42)	–	–
Antihistamines, n (%)	17 (14)	20 (17)	2 (13)	3 (13)	3 (11)	4 (17)	3 (14)	7 (19)	–	–

Hirschfield et al (2026) reported the duration of PBC and pruritus in months as median (interquartile range); Levy et al (2023) and Hegade et al (2017) reported them in years as mean (standard deviation). ALP and total bilirubin were reported in absolute units by Hirschfield et al (2026) and Hegade et al (2017) and as multiples of the upper limit of normal by Levy et al (2023); units are therefore not directly comparable across columns. A dash indicates that the variable was not reported.

ALP = alkaline phosphatase, BID = twice daily, IQR = interquartile range, PBC = primary biliary cholangitis, QD = once daily, SD = standard deviation, UDCA = ursodeoxycholic acid, ULN = upper limit of normal.

**Figure 1.**
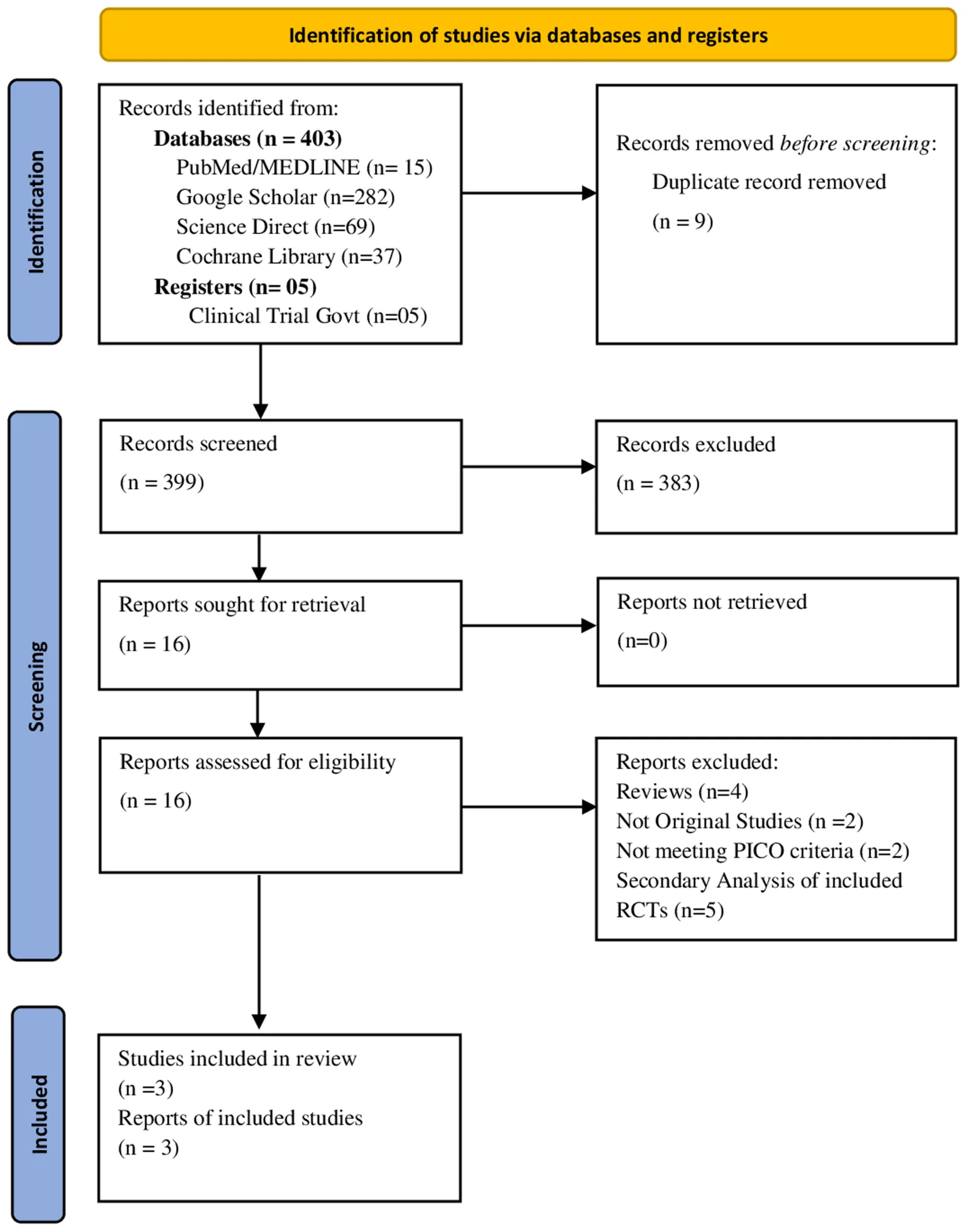
PRISMA 2020 flow diagram of study identification, screening, eligibility assessment, and inclusion. Of 408 records identified across 5 databases and registers, 3 randomized controlled trials met all eligibility criteria and were included in the quantitative synthesis. PRISMA — Preferred Reporting Items for Systematic Reviews and Meta-Analyses, RCT = randomised controlled trial.

The 3 included trials randomized 407 participants in total: 238 in the phase 3 GLISTEN trial,^[4]^ 147 in the phase 2b GLIMMER trial^[13]^ and 22 in the phase 2a crossover trial.^[12]^ Because the phase 2b trial compared 5 active dose arms against a single shared placebo group, and because only period 1 data were used from the crossover trial, the number of observations contributing to each analysis is smaller than the number of randomized participants and varies between outcomes according to data availability. Analysis-level denominators are stated for every outcome below and are displayed in the corresponding forest plots.

### 3.2. Risk of bias in included studies

The methodological quality of the 3 included RCTs was assessed using RoB 2.0. Hegade et al 2017, Levy et al 2023 and Hirschfield et al 2026 were judged to be at low risk of bias in all 5 domains assessed, namely bias arising from the randomization process, deviations from intended interventions, missing outcome data, measurement of the outcome, and selection of the reported result. All 3 studies therefore received an overall judgment of low risk of bias. The risk of bias is presented in Figures 2A, B and detailed in Table S2, Supplemental Digital Content 4.

**Figure 2.**
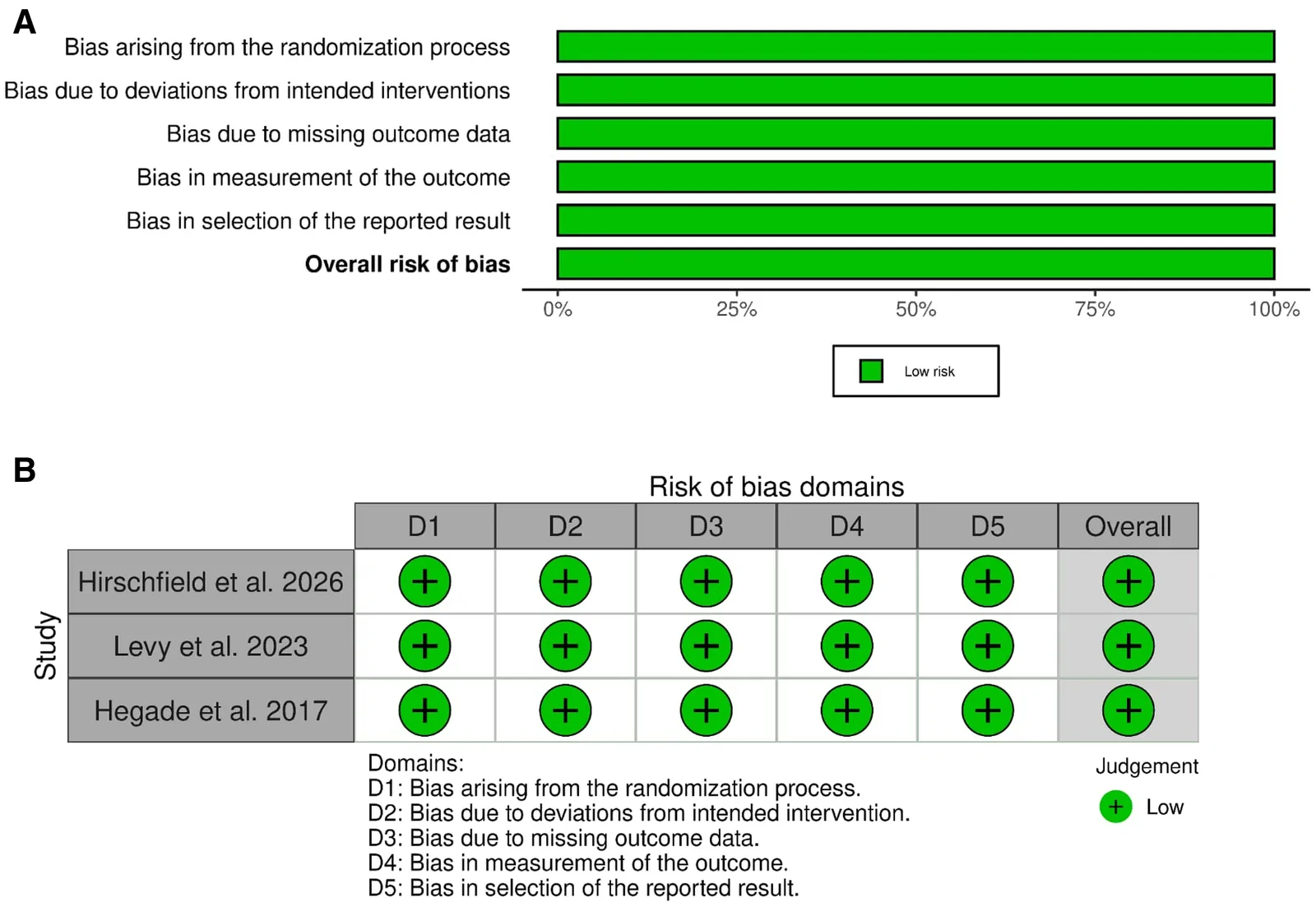
(A) Risk of bias graph. Reviewers’ judgements for each Cochrane Risk of Bias 2.0 domain, presented as percentages across the 3 included trials. (B) Risk of bias summary. Reviewers’ judgements for each Cochrane Risk of Bias 2.0 domain for each individual included trial.

### 3.3. Certainty of evidence

GRADE certainty was moderate for the primary WI-NRS outcome, downgraded once for imprecision because the pooled CI, although excluding no effect, lies entirely below the minimal clinically important difference (MCID). Certainty was moderate for adverse event discontinuation and for diarrhea, low for the responder thresholds, the PBC-40 itch domain, and the 5-D Itch Scale, and low for serum C4 and total bilirubin owing to serious inconsistency. The full summary-of-findings table is provided in Table S3, Supplemental Digital Content 2.

### 3.4. Primary outcome: effect on WI-NRS

Seven comparisons drawn from all 3 trials, comprising 231 participants allocated to linerixibat and 164 allocated to placebo, contributed to the primary analysis. Linerixibat produced a statistically significant reduction in WI-NRS score relative to placebo, with a pooled MD of −0.71 points (95% CI: −1.12 to −0.30; Z = 3.40; *P* = .0007). Between-study heterogeneity was absent (τ^2^ = 0.00; χ^2^ = 0.46, df = 6, *P* = 1.00; *I*^2^ = 0%), indicating a highly consistent effect across trial phases and dose regimens (Fig. 3). The magnitude of this pooled reduction is nonetheless smaller than the 2 to 3 point MCID established for chronic pruritus,^[22]^ and the entire 95% CI lies below that threshold; this is addressed in the Discussion.

**Figure 3.**
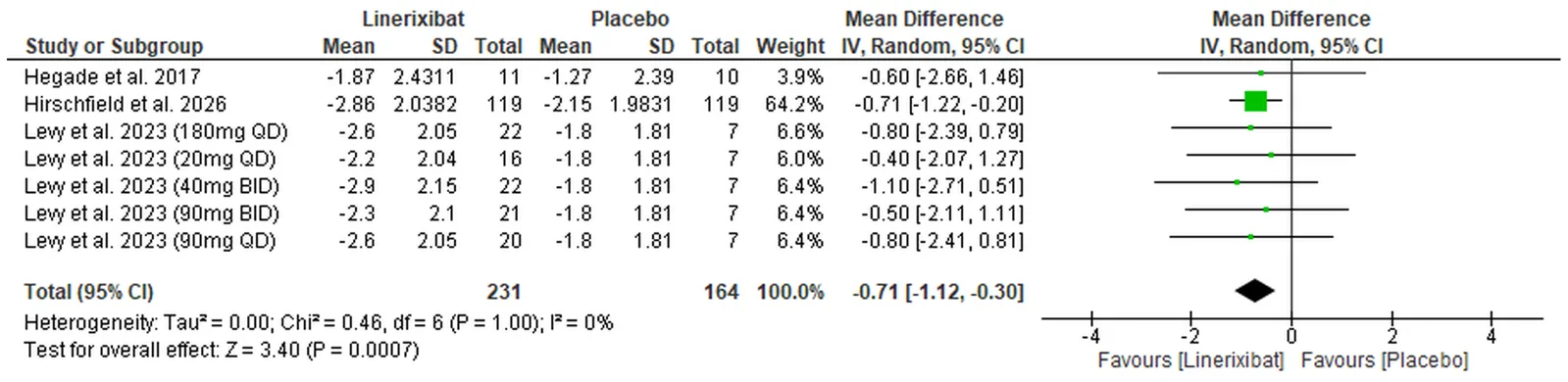
Forest plot of the change from baseline in Worst Itch Numerical Rating Scale (WI-NRS) score with linerixibat versus placebo. Seven comparisons from 3 trials (231 vs 164 participants). Pooled mean difference −0.71 (95% CI: −1.12 to −0.30); *P* = .0007; *I*^2^ = 0%. Random-effects inverse-variance model. BID = twice daily, CI = confidence interval, QD = once daily, SD = standard deviation.

### 3.5. Secondary efficacy outcomes

#### 3.5.1. Effect on PBC-40 itch domain score

Data from 2 trials, comprising 130 participants receiving linerixibat and 129 receiving placebo, were pooled. The MD favored linerixibat but did not reach statistical significance (MD −0.61; 95% CI: −1.41 to 0.19; *Z* = 1.49; *P* = .14), with no heterogeneity between studies (τ^2^ = 0.00; χ^2^ = 0.00, df = 1, *P* = .94; *I*^2^ = 0%; Fig. 4).

**Figure 4.**
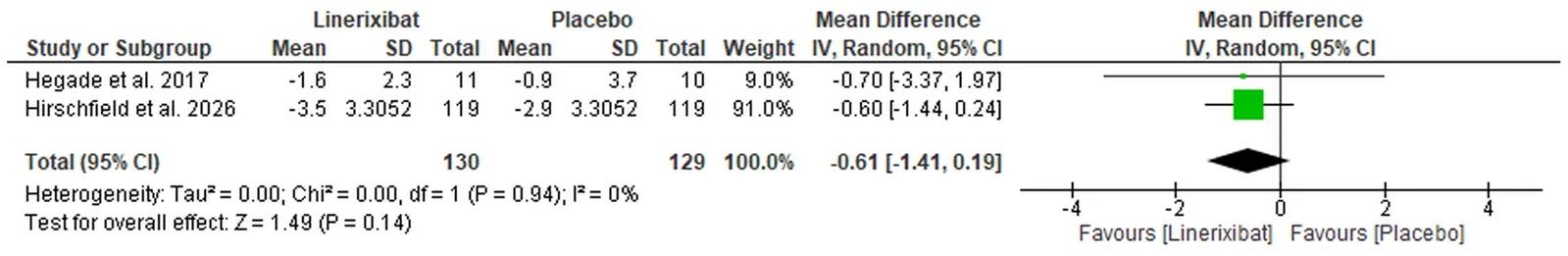
Forest plot of the change from baseline in PBC-40 itch domain score with linerixibat versus placebo. Two trials (130 vs 129 participants). Pooled mean difference −0.61 (95% CI −1.41 to 0.19); *P* = .14; *I*^2^ = 0%. Random-effects inverse-variance model. CI = confidence interval, SD = standard deviation.

#### 3.5.2. Effect on the 5-D itch scale

Six comparisons from 2 trials, comprising 111 participants receiving linerixibat and 45 receiving placebo, were pooled. No statistically significant difference was observed between groups (MD −0.77; 95% CI: −2.18 to 0.64; *Z* = 1.07; *P* = .28), with no heterogeneity (*I*^2^ = 0%; Fig. S3, Supplemental Digital Content 5).

Effect on pruritus-related sleep interference. Seven comparisons from all 3 trials, comprising 230 participants receiving linerixibat and 164 receiving placebo, were pooled. Linerixibat was associated with a small but statistically significant reduction in sleep interference relative to placebo (MD −0.47; 95% CI: −0.91 to −0.02; *Z* = 2.06; *P* = .04), with no heterogeneity (τ^2^ = 0.00; χ^2^ = 0.45, df = 6, *P* = 1.00; *I*^2^ = 0%; Fig. S6, Supplemental Digital Content 6).

### 3.6. Responder analyses

In accordance with the analytical approach described in the Methods, each responder threshold was analyzed as an independent meta-analysis, and no combined estimate across thresholds is presented. Six comparisons from 2 trials contributed to each analysis, comprising 230 participants receiving linerixibat and 155 receiving placebo.

For a reduction of ≥2 points in WI-NRS, 128 of 230 participants receiving linerixibat and 91 of 155 receiving placebo were responders; the pooled RR showed no significant difference (RR 1.06; 95% CI: 0.90–1.26; *Z* = 0.68; *P* = .49; *I*^2^ = 0%; Fig. S7, Supplemental Digital Content 7). For a reduction of ≥3 points, 121 of 230 versus 67 of 155 participants were responders, giving a borderline pooled effect that did not exclude no difference at the conventional threshold (RR 1.25; 95% CI: 1.00–1.56; *Z* = 1.96; *P* = .05; *I*^2^ = 0%; Fig. S8, Supplemental Digital Content 8). For a reduction of ≥4 points, 115 of 230 versus 55 of 155 participants were responders, with no statistically significant difference between groups (RR 1.23; 95% CI: 0.96–1.56; *Z* = 1.67; *P* = .10; *I*^2^ = 0%; Fig. S9, Supplemental Digital Content 9). Heterogeneity was absent at every threshold.

### 3.7. Pharmacodynamic biomarkers

#### 3.7.1. Serum FGF-19

Six comparisons from 2 trials, comprising 122 participants receiving linerixibat and 46 receiving placebo, showed a significant reduction in serum FGF-19 with linerixibat (MD −65.22 ng/L; 95% CI: −101.86 to −28.57; *Z* = 3.49; *P* = .0005), with no heterogeneity (τ^2^ = 0.00; χ^2^ = 1.31, df = 5, *P* = .93; *I*^2^ = 0%; Fig. 5).

**Figure 5.**
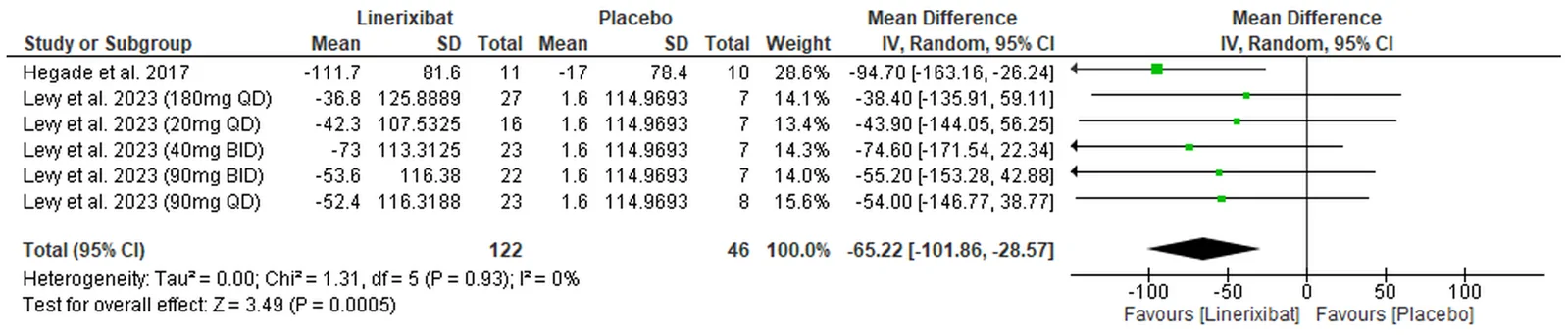
Forest plot of the change from baseline in serum fibroblast growth factor 19 (FGF-19, ng/L) with linerixibat versus placebo. Six comparisons from 2 trials (122 vs 46 participants). Pooled mean difference −65.22 (95% CI: −101.86 to −28.57); *P* = .0005; *I*^2^ = 0%. Random-effects inverse-variance model. BID = twice daily, CI = confidence interval, FGF-19 = fibroblast growth factor 19, QD = once daily, SD = standard deviation.

#### 3.7.2. Total serum bile acids

The same 6 comparisons (122 vs 46 participants) demonstrated a significant reduction in TSBA with linerixibat (MD −12.51 µmol/L; 95% CI: −20.72 to −4.29; *Z* = 2.98; *P* = .003), with no heterogeneity (χ^2^ = 1.65, df = 5, *P* = .89; *I*^2^ = 0%; Fig. S1, Supplemental Digital Content 10).

#### 3.7.3. Serum C4

Across the same 6 comparisons (122 vs 46 participants), linerixibat produced a significant increase in serum C4 concentration (MD 28.97 µmol/L; 95% CI: 17.10–40.85; *Z* = 4.78; *P* < .00001). Heterogeneity was high (τ^2^ = 190.20; χ^2^ = 37.74, df = 5, *P* < .00001; *I*^2^ = 87%), consistent with dose-dependent stimulation of compensatory bile acid synthesis across the dose-ranging arms (Fig. S2, Supplemental Digital Content 11). A leave-one-out sensitivity analysis was not informative because only 2 primary studies contributed data.

#### 3.7.4. Serum autotaxin

Because ATX was reported in different units and on different assay scales across trials, this outcome was pooled as a SMD. Six comparisons comprising 119 participants receiving linerixibat and 46 receiving placebo showed no statistically significant difference between groups (SMD −0.20; 95% CI: −0.55 to 0.15; *Z* = 1.11; *P* = .27), with no heterogeneity (τ^2^ = 0.00; χ^2^ = 1.35, df = 5, *P* = .93; *I*^2^ = 0%; Fig. S5, Supplemental Digital Content 12). This result differs in direction of inference from the individual trial reports and is discussed below.

### 3.8. Liver biochemistry

#### 3.8.1. Serum alkaline phosphatase

Two trials contributed data from 113 participants receiving linerixibat and 117 receiving placebo. No significant difference in ALP concentration was observed (MD 7.87 IU/L; 95% CI: −38.96 to 54.71; *Z* = 0.33; *P* = .74), with low heterogeneity (τ^2^ = 221.03; χ^2^ = 1.08, df = 1, *P* = .30; *I*^2^ = 7%; Fig. S4, Supplemental Digital Content 13).

#### 3.8.2. Total bilirubin

The same 2 trials (113 vs 117 participants) showed no significant difference in total bilirubin between groups (MD −1.07 µmol/L; 95% CI: −6.89 to 4.76; *Z* = 0.36; *P* = .72). Heterogeneity was substantial (τ^2^ = 13.57; χ^2^ = 3.69, df = 1, *P* = .05; *I*^2^ = 73%), reflecting opposite point estimates in the 2 contributing trials (Fig. 6). A leave-one-out sensitivity analysis could not be performed because data were available from only 2 studies.

**Figure 6.**
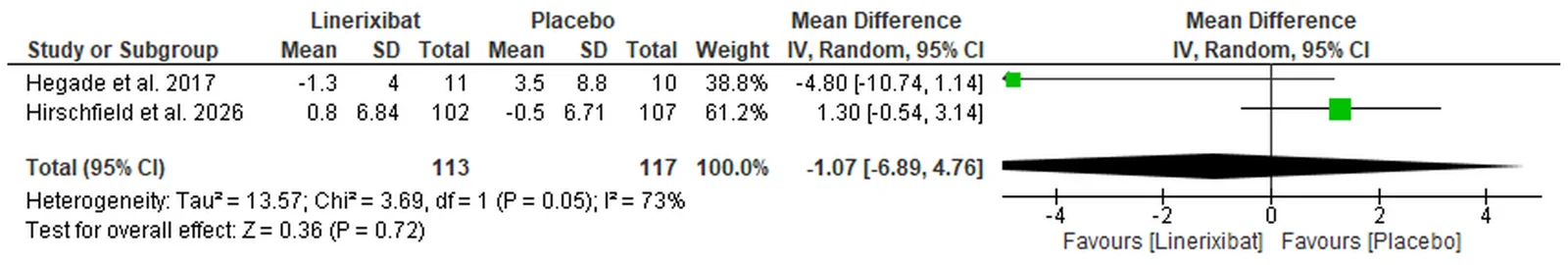
Forest plot of the change from baseline in total bilirubin (µmol/L) with linerixibat versus placebo. Two trials (113 vs 117 participants). Pooled mean difference −1.07 (95% CI: −6.89 to 4.76); *P* = .72; *I*^2^ = 73%. Random-effects inverse-variance model. CI = confidence interval, SD = standard deviation.

### 3.9. Safety outcomes

#### 3.9.1. Any adverse event

Seven comparisons from all 3 trials, comprising 251 exposures to linerixibat and 174 to placebo, were pooled. Adverse events occurred in 195 of 251 participants receiving linerixibat and 111 of 174 receiving placebo. The pooled RR favored placebo but did not reach statistical significance (RR 1.38; 95% CI: 0.99–1.93; *Z* = 1.88; *P* = .06), with moderate heterogeneity (τ^2^ = 0.06; χ^2^ = 11.82, df = 6, *P* = .07; *I*^2^ = 49%; Fig. S10, Supplemental Digital Content 14). Because heterogeneity fell below the prespecified 50% threshold after correction for the shared placebo group, the leave-one-out sensitivity analysis reported in the previous version of this synthesis was no longer required.

Adverse events leading to discontinuation of treatment. Six comparisons from 2 trials, comprising 230 participants receiving linerixibat and 153 receiving placebo, were pooled. Discontinuation occurred in 33 of 230 participants receiving linerixibat and 6 of 153 receiving placebo, giving a significantly increased risk with linerixibat (RR 2.75; 95% CI: 1.29–5.88; *Z* = 2.62; *P* = .009), with no heterogeneity (χ^2^ = 0.58, df = 4, *P* = .96; *I*^2^ = 0%; Fig. S11, Supplemental Digital Content 15).

Adverse events occurring in ≥5% of patients. Six comparisons from 2 trials, comprising 230 participants receiving linerixibat and 154 receiving placebo, showed a significantly greater risk of adverse events reported in at least 5% of participants (189 of 230 vs 79 of 154; RR 1.39; 95% CI: 1.12–1.73; *Z* = 2.99; *P* = .003), with low heterogeneity (τ^2^ = 0.02; χ^2^ = 6.90, df = 5, *P* = .23; *I*^2^ = 28%; Fig. S12, Supplemental Digital Content 16).

#### 3.9.2. Individual adverse events

A subgroup analysis of eleven individual adverse events across the 3 trials confirmed an overall excess risk with linerixibat (RR 2.00; 95% CI: 1.61–2.49; *Z* = 6.23; *P* < .00001; *I*^2^ = 0%), with significant differences between event subgroups (χ^2^ = 26.66, df = 10; *P* = .003; *I*^2^ = 62.5%). The excess was confined to gastrointestinal events. Diarrhea was the dominant adverse event (145 of 251 vs 26 of 175; RR 3.69; 95% CI: 2.55–5.32; *P* < .00001; *I*^2^ = 0%), followed by abdominal pain (44 of 251 vs 7 of 175; RR 2.80; 95% CI: 1.33–5.90; *P* = .007; *I*^2^ = 3%) and dyspepsia (RR 7.22; 95% CI: 1.63–32.09; *P* = .009; *I*^2^ = 0%). No significant excess was observed for nausea (RR 1.18; 95% CI: 0.61–2.30; *P* = .62), headache (RR 1.33; 95% CI: 0.71–2.48; *P* = .38), constipation (RR 2.43; 95% CI: 0.15–40.65; *P* = .54; *I*^2^ = 73%), abdominal distension (RR 1.53; 95% CI: 0.61–3.89; *P* = .37), upper abdominal pain (RR 1.74; 95% CI: 0.74–4.06; *P* = .20), dizziness (RR 1.33; 95% CI: 0.46–3.88; *P* = .60), nasopharyngitis (RR 0.80; 95% CI: 0.23–2.73; *P* = .72), or fatigue (RR 0.54; 95% CI: 0.18–1.62; *P* = .27; Fig. S13, Supplemental Digital Content 17).

### 3.10. Dose-specific analysis: linerixibat 40 mg twice daily

A dedicated analysis restricted to the licensed 40 mg twice-daily regimen, comprising the phase 3 trial and the corresponding arm of the phase 2b trial, produced results closely concordant with the primary synthesis. The WI-NRS score was significantly reduced (141 vs 126 participants; MD −0.75; 95% CI: −1.23 to −0.26; *Z* = 3.00; *P* = .003; *I*^2^ = 0%; Fig. S14, Supplemental Digital Content 3), as was pruritus-related sleep interference (MD −0.53; 95% CI: −1.04 to −0.02; *Z* = 2.02; *P* = .04; *I*^2^ = 0%; Fig. S15, Supplemental Digital Content 18). Responder rates were not significantly increased at the ≥2-point threshold (RR 1.05; 95% CI: 0.88–1.26; *P* = .57), were borderline at ≥3 points (RR 1.28; 95% CI: 1.00–1.65; *Z* = 1.94; *P* = .05), and were not significant at ≥4 points (RR 1.33; 95% CI: 0.97–1.82; *Z* = 1.76; *P* = .08; Figs. S16, S17 and S18, Supplemental Digital Content 19). Overall adverse events were not significantly increased (RR 1.61; 95% CI: 0.59–4.42; *P* = .36; *I*^2^ = 43%; Fig. S19, Supplemental Digital Content 20), nor were adverse events occurring in ≥5% of patients (RR 1.42; 95% CI: 0.87–2.33; *P* = .17; *I*^2^ = 69% Fig. S21, Supplemental Digital Content 21), whereas discontinuation for adverse events remained significantly more frequent with linerixibat (18 of 142 vs 6 of 125; RR 2.59; 95% CI: 1.10–6.13; *Z* = 2.17; *P* = .03; *I*^2^ = 0%; Fig. S20, Supplemental Digital Content 22). The consistency of the dose-specific and all-dose estimates indicates that pooling across the dose-ranging arms did not materially distort the estimate of effect for the regimen in clinical use.

## 4. Discussion

This systematic review and meta-analysis synthesized data from 3 RCTs of linerixibat, an IBAT inhibitor, in adults with PBC-associated cholestatic pruritus: Hegade et al 2017, Levy et al 2023 (GLIMMER), and Hirschfield et al 2026 (GLISTEN). The pooled data show a statistically significant but numerically small reduction in itch intensity and sleep interference, unambiguous pharmacodynamic evidence of target engagement, and a clear gastrointestinal tolerability burden that translates into a substantially increased rate of treatment discontinuation. Interpreting these findings requires that the statistical significance of the primary outcome and its clinical magnitude be considered separately.

### 4.1. Statistical significance and clinical magnitude

The pooled reduction in WI-NRS of −0.71 points (95% CI: −1.12 to −0.30) was highly consistent across trials (*I*^2^ = 0%) and statistically robust (*P* = .0007). It is nonetheless smaller than the MCID for chronic pruritus, which anchor-based studies place at 2 to 3 points on a 0 to 10 NRS,^[22]^ and the upper confidence limit of our estimate remains well below that threshold. On the available evidence, therefore, the average patient treated with linerixibat should not be expected to experience a change in itch intensity that they themselves would recognize as clinically meaningful. We have accordingly removed the characterization of linerixibat as a potent targeted therapy from the abstract and conclusion of this manuscript, as that description overstated the magnitude of the observed effect.

Three considerations qualify, without overturning, this conservative reading. First, a MD between groups is not the same quantity as a within-patient change: the within-patient reduction from baseline in the phase 3 trial was −2.86 points, and the MCID was derived for within-patient change, so the between-group difference of −0.71 represents the placebo-subtracted increment rather than the total symptomatic improvement a patient experiences. Second, a mean effect may conceal a clinically meaningful response in a subgroup of patients; this is the rationale for responder analyses. Third, however, our responder analyses provide only equivocal support for that possibility. When the 3 cumulative thresholds are analyzed separately, as they must be, the effect at ≥3 points is borderline (RR 1.25; 95% CI: 1.00–1.56; *P* = .05) and the effects at ≥2 and ≥4 points are not statistically significant. The previously reported combined estimate across thresholds is not a valid summary, because the thresholds are nested within the same cohort and pooling them counts individual participants up to 3 times; it has been removed from this revision.

### 4.2. Discordance between itch instruments

The statistically significant effect on WI-NRS was not reproduced on the 5-D Itch Scale (MD −0.77; *P* = .28) or the PBC-40 itch domain (MD −0.61; *P* = .14). This discordance has several plausible explanations and does not by itself invalidate the primary finding. The WI-NRS is a single-item, unidimensional daily measure of worst itch intensity with a 24-hour recall, and it is the instrument that has been formally validated and shown to be responsive in this specific population.^[23]^ The 5-D Itch Scale is a multidimensional composite that incorporates duration, degree, direction, disability, and distribution, so a change confined to intensity is diluted across 4 other dimensions that IBAT inhibition would not be expected to alter within the trial period. The PBC-40 itch domain is embedded within a broader HRQoL instrument, uses a different scoring metric and a longer recall period, and is therefore less specific to short-term change in itch intensity.^[24]^ Statistical power differs substantially as well: 395 observations contributed to the WI-NRS analysis compared with 156 for the 5-D Itch Scale and 259 for the PBC-40 itch domain, and the phase 3 trial contributed no 5-D Itch data at all. The direction of effect was consistent in favor of linerixibat on all 3 instruments, with 0 heterogeneity throughout. The most defensible interpretation is that the effect of linerixibat is real but small and is detectable only on the most sensitive and best-powered instrument. It would not be appropriate to present the WI-NRS result as though the secondary itch instruments corroborated it.

### 4.3. Mechanism and pharmacodynamic findings

Linerixibat acts by inhibiting the apical sodium-dependent bile acid transporter (ASBT/SLC10A2) in the ileal enterocyte, interrupting the enterohepatic circulation and increasing fecal bile acid excretion.^[25]^ Cholestatic pruritus is thought to involve pruritogenic substances that accumulate in the systemic circulation during cholestasis, particularly lysophosphatidic acid (LPA), which is generated by the enzyme ATX and binds unmyelinated C fibers in the skin.^[26,27]^

The pharmacodynamic findings of this synthesis confirm target engagement. The marked reduction in TSBA and in FGF-19, together with the marked increase in serum C4, are internally consistent and mechanistically coherent. FGF-19 is an ileal hormone released in response to bile acid uptake through farnesoid X receptor activation, and its reduction indicates successful inhibition of ileal bile acid uptake; C4 is an index of hepatic bile acid synthesis and rises as a compensatory response to interruption of the enterohepatic circulation.^[28]^ The high heterogeneity observed for C4 (*I*^2^ = 87%) is explicable by the dose-ranging design of the phase 2b trial, in which the compensatory synthetic response scaled with dose from a MD of 8.40 in the 20 mg once-daily arm to 49.20 in the 40 mg twice-daily arm.

The ATX result requires particular candor. Serum ATX is an established biomarker of cholestatic pruritus, is elevated in cholestatic but not non-cholestatic itch, and has been reported to fall in parallel with symptomatic improvement.^[25,29]^ In the present synthesis, however, pooling ATX as a SMD across trials that reported it in different units yielded no significant effect (SMD −0.20; 95% CI: −0.55 to 0.15; *P* = .27). We therefore cannot claim, as an earlier version of this manuscript did, that linerixibat significantly lowers ATX. The point estimate favors linerixibat and heterogeneity was absent, so the finding is best read as an underpowered null rather than as positive evidence of no effect: only 165 observations contributed, and standardization across heterogeneous assays introduces additional noise. The mechanistic case for IBAT inhibition in cholestatic pruritus rests, on our data, on the bile acid axis (TSBA, FGF-19, and C4) rather than on demonstrated modulation of the ATX-LPA axis.

The absence of any effect on ALP or total bilirubin supports the view that linerixibat acts on the symptomatic pathway without modifying the underlying cholestatic process, consistent with previous observations for IBAT inhibitors.^[30]^ This is an important point for clinical communication: linerixibat is a symptomatic therapy and should not be presented to patients as disease-modifying.

### 4.4. Balancing benefit against harm

A balanced assessment of linerixibat must weigh a modest average benefit against a real and frequent tolerability burden. In this synthesis, the overall risk of experiencing any adverse event was not significantly increased (RR 1.38; 95% CI: 0.99–1.93; *P* = .06). This is a change from the previous version of this analysis, in which the corresponding estimate was significant; the difference is attributable entirely to correcting the shared placebo group of the phase 2b trial, which had previously inflated the control denominator and narrowed the CI. The reviewer’s observation that the earlier adverse-event result depended heavily on a single trial was therefore well-founded, and the corrected analysis no longer supports a claim of significantly increased overall adverse event risk.

What the corrected analysis does support is a specific and clinically important gastrointestinal signal. Diarrhea occurred in 145 of 251 participants receiving linerixibat compared with 26 of 175 receiving placebo (RR 3.69; 95% CI: 2.55–5.32), abdominal pain in 44 versus 7 (RR 2.80), and dyspepsia was also significantly increased (RR 7.22). These effects are the predictable pharmacological consequence of increased colonic bile acid delivery and resultant osmotic diarrhea.^[26]^ Critically, these events are not merely nuisance effects: discontinuation of treatment because of adverse events was approximately 3 times more frequent with linerixibat than with placebo (RR 2.75; 95% CI: 1.29–5.88; *P* = .009), a finding that was consistent at the licensed 40 mg twice-daily dose (RR 2.59; 95% CI: 1.10–6.13; *P* = .03) and that carried no heterogeneity in either analysis.

The clinical implication follows directly from placing these 2 findings side by side. For the average patient, linerixibat offers a placebo-subtracted reduction in itch intensity below the recognized threshold for a perceptible change, together with an approximately threefold probability of stopping treatment because of adverse effects, most commonly diarrhea. This is not a favorable ratio in unselected patients, and linerixibat should not be positioned as a first line or routine therapy for cholestatic pruritus. It is, however, a reasonable and now licensed option in the specific population in whom it was studied and for whom alternatives are genuinely absent: patients with moderate-to-severe pruritus that has proved refractory to cholestyramine and to off-label second line agents. In that group, in whom intractable itch is a recognized indication for liver transplantation irrespective of hepatic function^[31]^ and is associated with substantially increased suicide risk, a modest average benefit may still be worth pursuing, provided that the expected magnitude of benefit and the high likelihood of diarrhea are discussed explicitly and that treatment is discontinued if no meaningful response occurs. Response should be assessed against a pre-agreed individual threshold rather than assumed.

The dose-specific analysis is reassuring in this respect. Estimates for the licensed 40 mg BID regimen were closely concordant with the all-dose synthesis for efficacy (WI-NRS MD−0.75 vs −0.71) while showing no significant excess of overall adverse events, which supports the dose selected for phase 3 as offering the most acceptable balance of efficacy and tolerability and indicates that the higher exploratory doses used in the phase 2b trial contributed disproportionately to the gastrointestinal burden observed across the dose-ranging arms.

## 5. Limitations

This review has several limitations, some of which are intrinsic to the small evidence base and some of which reflect analytical decisions that we consider it important to state explicitly.

First, only 3 trials were eligible, and they differed in phase, design, and duration. This precluded meta-regression, subgroup analysis beyond the prespecified dose-specific comparison, and any formal assessment of publication bias; funnel plot asymmetry and Egger’s test are not interpretable at *k* = 3. Publication bias should therefore be regarded as undetected rather than excluded. For outcomes informed by only 2 studies, including serum C4, total bilirubin, and ALP, leave-one-out sensitivity analysis was not informative, and the substantial heterogeneity observed for C4 (*I*^2^ = 87%) and total bilirubin (*I*^2^ = 73%) could not be explored analytically.

Second, the choice of the DerSimonian–Laird estimator over the HKSJ adjustment, justified in the Methods, means that CIs for the small number of outcomes with nonzero heterogeneity may be narrower than a fully conservative small-sample estimator would produce. For the primary outcome and for the majority of secondary outcomes, where *I*^2^ = 0% and τ^2^ = 0.00, this choice has no material effect on the intervals reported.

Third, a residual unit-of-analysis limitation remains in the safety analyses. Efficacy and biomarker outcomes from the phase 2a crossover trial were restricted to period 1 data, which entirely removes carryover and period effects. Adverse events in that trial, however, are reported by treatment received across both periods, and period-specific adverse event data could not be recovered from the published report; the same 21 participants therefore contribute to both arms of the safety analyses. This paired structure is not accounted for in the pooled safety estimates. The affected trial carries 34.2% weight in the any adverse event analysis and contributes to the individual event subgroups, so this limitation should be borne in mind when interpreting those estimates, although it does not affect the discontinuation analysis, to which the phase 2a trial did not contribute.

Fourth, the pooled efficacy estimate combines dose arms ranging from 20 mg QD to 90 mg BID. Although the dedicated 40 mg BID analysis was concordant with the all-dose estimate, the all-dose synthesis remains a summary across regimens that are not clinically interchangeable, and the dose-specific analysis is itself limited to 2 contributing arms.

Fifth, ATX was pooled as a SMD because of unit heterogeneity across trials, which reduces interpretability and statistical power relative to a raw MD; the null result for this outcome should not be read as evidence of absence of an effect.

Finally, evidence on long-term safety and efficacy beyond 24 weeks is scarce, and no included trial reported outcomes beyond that horizon. Future research should address long-term tolerability, real-world adherence at the licensed dose, whether the subgroup of patients who achieve a clinically meaningful response can be identified prospectively, and strategies to mitigate gastrointestinal adverse effects.

## 6. Conclusion

Linerixibat, the first medicine approved by the Food and Drug Administration specifically for cholestatic pruritus in PBC, produces a statistically significant and highly consistent reduction in itch intensity together with clear pharmacodynamic evidence of target engagement along the bile acid axis. The magnitude of the average effect is modest and falls below the accepted MCID for chronic pruritus, the effect is not reproduced on secondary itch instruments, and responder benefit is borderline at the ≥3-point threshold and absent at ≥2 and ≥4 points. These benefits must be set against a marked excess of gastrointestinal adverse effects, principally diarrhea, and an approximately threefold increase in treatment discontinuation. Linerixibat is therefore best regarded as a mechanistically validated option for patients with moderate-to-severe cholestatic pruritus refractory to existing therapy, to be initiated with explicit discussion of the expected magnitude of benefit and the likelihood of gastrointestinal intolerance, and to be discontinued in the absence of a meaningful individual response. Larger and longer trials, and analyses capable of identifying responders prospectively, are required before its place in routine practice can be defined.

## Author contributions

**Conceptualization:** Mukesh Kumar.

**Data curation:** Mukesh Kumar.

**Formal analysis:** Mukesh Kumar, Mahwish Gulbahar.

**Investigation:** Mahwish Gulbahar.

**Methodology:** Mukesh Kumar, Laksh Kumar, FNU Poonam.

**Project administration:** Vivek Kumar.

**Software:** Vivek Kumar, Usba Rafique, Um E Farwa, FNU Poonam.

**Supervision:** Sumet Kumar, Aayush Chaulagain.

**Validation:** Sumet Kumar, Um E Farwa.

**Writing – review & editing:** Vivek Kumar, Syeda Warisha, Munazzah Khan, Rehma Binte Nasir, FNU Bakhtawar.

**Writing – original draft:** Syeda Warisha, Munazzah Khan, Savera Shabbir, Laksh Kumar, Rehma Binte Nasir, Murk Kumari Pamnani, Surhan Indra, FNU Bakhtawar.
















































