# Supplementary material for: The role of linerixibat in treating pruritus in primary biliary cholangitis: A systematic review and meta-analysis of randomised controlled trials
Source: Medicine (Baltimore). 2026 Oct 30;105(44):e51203. doi: 10.1097/MD.0000000000051203 (PMC13727225; doi:10.1097/MD.0000000000051203)
Supplement: Supplementary file 1 [file medi-105-e51203-s001.docx]

The Role of Linerixibat in Treating Pruritus in Primary Biliary Cholangitis: A Systematic Review and Meta-Analysis of Randomised Controlled Trials

Note: In accordance with journal requirements, all supplementary figures (S1–S21) are supplied as separate individual image files and are not embedded in this document. Legends for all supplementary tables and figures appear at the end of the main manuscript file.

**Supplementary Table S1. Search Strategy**

| **Database / Register** | **Records retrieved** | **Search string** |
| --- | --- | --- |
| PubMed/MEDLINE | 15 | "Linerixibat"[All Fields] AND ("pruritus"[MeSH Terms] OR "pruritus"[All Fields]) AND ("liver cirrhosis, biliary"[MeSH Terms] OR ("liver"[All Fields] AND "cirrhosis"[All Fields] AND "biliary"[All Fields]) OR "biliary liver cirrhosis"[All Fields] OR ("primary"[All Fields] AND "biliary"[All Fields] AND "cholangitis"[All Fields]) OR "primary biliary cholangitis"[All Fields]) |
| Cochrane Library (CENTRAL) | 37 | (Linerixibat) AND (pruritus) AND (primary biliary cholangitis) |
| Google Scholar | 282 | (Linerixibat) AND (pruritus) AND (primary biliary cholangitis) |
| ScienceDirect | 69 | (Linerixibat) AND (pruritus) AND (primary biliary cholangitis) |
| ClinicalTrials.gov | 5 | (Linerixibat) AND (pruritus) AND (primary biliary cholangitis) |
| **Total** | **408** | **Nine duplicates removed; 399 records screened** |

Searches were run from database inception to April 2026 and restricted to English language publications. Reference lists of included trials and relevant systematic reviews were screened manually.
