# Supplementary material for: The role of linerixibat in treating pruritus in primary biliary cholangitis: A systematic review and meta-analysis of randomised controlled trials
Source: Medicine (Baltimore). 2026 Oct 30;105(44):e51203. doi: 10.1097/MD.0000000000051203 (PMC13727225; doi:10.1097/MD.0000000000051203)
Supplement: Supplementary file 2 [file medi-105-e51203-s002.docx]

**Supplementary Table S3. GRADE Summary of Findings**

Linerixibat compared with placebo for cholestatic pruritus in primary biliary cholangitis

| **Outcome** | **Participants (studies)** | **Pooled effect (95% CI)** | **Certainty** | **Reasons for downgrading** |
| --- | --- | --- | --- | --- |
| WI-NRS change from baseline (primary) | 395 (3 RCTs) | MD −0.71 (−1.12 to −0.30) | ⊕⊕⊕⊖ Moderate | Imprecision (−1): although the CI excludes no effect, it lies entirely below the 2–3 point MCID, so the estimate cannot establish a clinically meaningful benefit. |
| Pruritus-related sleep interference | 394 (3 RCTs) | MD −0.47 (−0.91 to −0.02) | ⊕⊕⊕⊖ Moderate | Imprecision (−1): upper CI limit approaches no effect and the estimate is small relative to the scale range. |
| PBC-40 itch domain score | 259 (2 RCTs) | MD −0.61 (−1.41 to 0.19) | ⊕⊕⊖⊖ Low | Imprecision (−2): CI includes both no effect and appreciable benefit; few participants and only two contributing trials. |
| 5-D Itch Scale score | 156 (2 RCTs) | MD −0.77 (−2.18 to 0.64) | ⊕⊕⊖⊖ Low | Imprecision (−2): wide CI spanning no effect; the phase 3 trial contributed no data to this outcome. |
| Responder rate, ≥2-point WI-NRS reduction | 385 (2 RCTs) | RR 1.06 (0.90 to 1.26) | ⊕⊕⊖⊖ Low | Imprecision (−2): CI includes both appreciable benefit and appreciable harm; few events outside the phase 3 trial. |
| Responder rate, ≥3-point WI-NRS reduction | 385 (2 RCTs) | RR 1.25 (1.00 to 1.56) | ⊕⊕⊖⊖ Low | Imprecision (−2): lower CI limit touches the line of no effect (P = 0.05); estimate is dominated by a single trial. |
| Responder rate, ≥4-point WI-NRS reduction | 385 (2 RCTs) | RR 1.23 (0.96 to 1.56) | ⊕⊕⊖⊖ Low | Imprecision (−2): CI includes no effect; analysis is underpowered at this threshold. |
| Total serum bile acids | 168 (2 RCTs) | MD −12.51 µmol/L (−20.72 to −4.29) | ⊕⊕⊕⊖ Moderate | Indirectness (−1): pharmacodynamic surrogate rather than a patient-important outcome. |
| Serum FGF-19 | 168 (2 RCTs) | MD −65.22 ng/L (−101.86 to −28.57) | ⊕⊕⊕⊖ Moderate | Indirectness (−1): pharmacodynamic surrogate rather than a patient-important outcome. |
| Serum C4 | 168 (2 RCTs) | MD 28.97 µmol/L (17.10 to 40.85) | ⊕⊕⊖⊖ Low | Indirectness (−1): pharmacodynamic surrogate. Inconsistency (−1): I² = 87%, attributable to dose-dependent compensatory bile acid synthesis across dose-ranging arms. |
| Serum autotaxin | 165 (2 RCTs) | SMD −0.20 (−0.55 to 0.15) | ⊕⊕⊖⊖ Low | Indirectness (−1): pharmacodynamic surrogate, pooled as SMD across differing assay units. Imprecision (−1): CI includes no effect and appreciable benefit. |
| Serum alkaline phosphatase | 230 (2 RCTs) | MD 7.87 IU/L (−38.96 to 54.71) | ⊕⊕⊖⊖ Low | Indirectness (−1): biochemical surrogate. Imprecision (−2, capped at −1 with indirectness): very wide CI spanning clinically opposite effects. |
| Total bilirubin | 230 (2 RCTs) | MD −1.07 µmol/L (−6.89 to 4.76) | ⊕⊕⊖⊖ Low | Inconsistency (−1): I² = 73% with opposite point estimates in the two trials. Imprecision (−1): CI spans no effect. |
| Any adverse event | 425 (3 RCTs) | RR 1.38 (0.99 to 1.93) | ⊕⊕⊖⊖ Low | Risk of bias (−1): the phase 2a crossover trial contributes paired within-patient safety data to both arms. Imprecision (−1): CI includes no effect. |
| Adverse event leading to discontinuation | 383 (2 RCTs) | RR 2.75 (1.29 to 5.88) | ⊕⊕⊕⊖ Moderate | Imprecision (−1): only 39 events in total, producing a wide CI despite a large point estimate. |
| Diarrhoea | 426 (3 RCTs) | RR 3.69 (2.55 to 5.32) | ⊕⊕⊕⊖ Moderate | Risk of bias (−1): the phase 2a crossover trial contributes paired within-patient safety data to both arms. Effect is large and consistent (I² = 0%). |
| Abdominal pain | 426 (3 RCTs) | RR 2.80 (1.33 to 5.90) | ⊕⊕⊕⊖ Moderate | Imprecision (−1): relatively few events (51 in total) with a wide CI. |

GRADE Working Group certainty of evidence. High (⊕⊕⊕⊕): very confident the true effect lies close to the estimate. Moderate (⊕⊕⊕⊖): moderately confident; the true effect is likely close but may be substantially different. Low (⊕⊕⊖⊖): confidence is limited; the true effect may be substantially different. Very low (⊕⊖⊖⊖): very little confidence.

All outcomes derive from randomised controlled trials and therefore began at high certainty. Publication bias could not be assessed statistically because only three trials were available; it is recorded as undetected rather than absent for every outcome and was not used as a basis for downgrading. Participant counts are analysis-level denominators after correction for the shared placebo group and restriction of the crossover trial to period 1 data, and therefore differ from the 407 randomised participants.

Abbreviations: C4, 7α-hydroxy-4-cholesten-3-one; CI, confidence interval; FGF-19, fibroblast growth factor 19; GRADE, Grading of Recommendations, Assessment, Development and Evaluations; MCID, minimal clinically important difference; MD, mean difference; PBC-40, primary biliary cholangitis 40-item quality of life instrument; RCT, randomised controlled trial; RR, risk ratio; SMD, standardised mean difference; WI-NRS, Worst Itch Numerical Rating Scale.
