# Supplementary material for: The role of linerixibat in treating pruritus in primary biliary cholangitis: A systematic review and meta-analysis of randomised controlled trials
Source: Medicine (Baltimore). 2026 Oct 30;105(44):e51203. doi: 10.1097/MD.0000000000051203 (PMC13727225; doi:10.1097/MD.0000000000051203)
Supplement: Supplementary file 3 [file medi-105-e51203-s004.docx]

**Supplementary Table S2. Risk of Bias Assessment (Cochrane RoB 2.0)**

| **Study** | **Domain** | **Judgement** | **Justification** |
| --- | --- | --- | --- |
| Hegade et al. 2017 (BAT117213; NCT01899703) | Randomisation process | Low risk | Central computer-generated sequence (RandAll) with 1:1 allocation and block size of 4; allocation concealment maintained. |
|  | Deviations from intended interventions | Low risk | Double-blind crossover design with successful masking of participants and staff; no significant deviations reported. |
|  | Missing outcome data | Low risk | 21 of 22 randomised patients completed all procedures; the single withdrawal occurred before treatment and does not bias the results. |
|  | Measurement of the outcome | Low risk | Double-blinding maintained with identical placebos; no direct evidence that gastrointestinal side effects unblinded subjective pruritus scoring. |
|  | Selection of the reported result | Low risk | Prospectively registered (NCT01899703); outcomes reported according to the pre-specified analysis plan. |
|  | Overall | Low risk | Well-designed trial with robust randomisation, concealment and blinding. |
| Levy et al. 2023 (GLIMMER; NCT02966834) | Randomisation process | Low risk | Central randomisation (3:1) with stratification by region and bilirubin level, ensuring adequate sequence generation and concealment. |
|  | Deviations from intended interventions | Low risk | Double-blind design with identical placebos; primary analysis used the intention-to-treat population including all randomised patients receiving at least one dose. |
|  | Missing outcome data | Low risk | Some participants withdrew because of gastrointestinal adverse events, predominantly in high-dose groups; mixed-model repeated measures used to handle missing data appropriately. |
|  | Measurement of the outcome | Low risk | Outcome measured with a validated 0–10 NRS eDiary by blinded participants; double-blinding maintained despite known gastrointestinal side effects. |
|  | Selection of the reported result | Low risk | Prospectively registered (NCT02966834); outcomes reported according to the pre-specified statistical analysis plan. |
|  | Overall | Low risk | Well-conducted multicentre RCT with robust methodology and appropriate statistical handling of patient data. |
| Hirschfield et al. 2026 (GLISTEN; NCT04950127) | Randomisation process | Low risk | Central randomisation (1:1) via interactive response technology with stratification by pruritus severity ensured allocation concealment. |
|  | Deviations from intended interventions | Low risk | Double-blind trial with identical placebos and no significant deviations reported; analysis followed the intention-to-treat principle. |
|  | Missing outcome data | Low risk | Discontinuations were balanced between arms (14% linerixibat vs 13% placebo) and handled using appropriate multiple imputation methods for the primary analysis. |
|  | Measurement of the outcome | Low risk | Pruritus measured using a validated 0–10 NRS eDiary; blinding remained intact despite high rates of gastrointestinal side effects. |
|  | Selection of the reported result | Low risk | Results reported in accordance with the pre-registered protocol (NCT04950127) and the statistical analysis plan. |
|  | Overall | Low risk | Phase 3 trial of high methodological quality with robust blinding and appropriate handling of missing data. |

Abbreviations: eDiary, electronic diary; NRS, numerical rating scale; RCT, randomised controlled trial; RoB 2.0, Cochrane Risk of Bias 2.0 tool.

Note: the ClinicalTrials.gov identifier for the GLISTEN trial has been corrected to NCT04950127 in this revision.
